# FGF21 increases water intake, urine output and blood pressure in rats

**DOI:** 10.1371/journal.pone.0202182

**Published:** 2018-08-14

**Authors:** Tod Turner, Xian Chen, Matthew Zahner, Alan Opsahl, George DeMarco, Magalie Boucher, Bryan Goodwin, Mylène Perreault

**Affiliations:** 1 Internal Medicine Research Unit, Pfizer Inc, Cambridge, Massachusetts, United States of America; 2 Comparative Medicine, Pfizer Inc, Cambridge, Massachusetts, United States of America; 3 Drug Safety Research and Development (DSRD), Pfizer Inc, Groton, Connecticut, United States of America; 4 Drug Safety Research and Development (DSRD), Pfizer Inc, Cambridge, Massachusetts, United States of America; Hopital Tenon, FRANCE

## Abstract

Fibroblast growth factor 21 (FGF21) is a hormone secreted by the liver in response to metabolic stress. In addition to its well-characterized effects on energy homeostasis, FGF21 has been shown to increase water intake in animals. In this study, we sought to further explore the effects of FGF21 on fluid homeostasis in rats. A single dose of a long-acting FGF21 analog, PF-05231023, significantly increased water consumption, which was accompanied by an elevation in urine output that appeared prior to a significant change in water intake. We observed that FGF21 rapidly and significantly increased heart rate and blood pressure in telemeter-implanted rats, before changes in urine output and water intake were observed. Our data suggest that sympathetic activation may contribute to the pathogenesis by which FGF21 increases blood pressure as the baroreceptor unloading induced reflex tachycardia was significantly elevated in FGF21-treated animals. However, FGF21 was still capable of causing hypertension in animals in which approximately 40% of the sympathetic post-ganglionic neurons were ablated. Our data suggest that FGF21-induced water intake is in fact secondary to diuresis, which we propose to be a compensatory mechanism engaged to alleviate the acute hypertension caused by FGF21.

## Introduction

Fibroblast growth factor 21 (FGF21) is a peptide hormone secreted by the liver that modulates metabolism and growth in response to metabolic stress. The effects of FGF21 are mediated by binding to β-klotho (KLB) and its preferred isoform of the FGF receptor, FGFR1c [1, 2]. Activation of KLB/FGFR1c by native or synthetic FGF21 molecules has been shown to induce body weight loss and reduce circulating triglyceride levels in rodent models of obesity, non-human primates and humans [1, 3–6]. The pharmacological effects of FGF21 on metabolism are believed to be mediated in part by directly modulating catabolic/anabolic pathways in white and brown adipose tissues, and indirectly by acting in the central nervous system (CNS), specifically the hypothalamus and dorsal vagal complex [7–10]. Although the exact mechanism by which FGF21 centrally regulates metabolic parameters is not fully understood, several reports indicate that the hypothalamic pituitary adrenal (HPA) axis and sympathetic activation are involved [7–9, 11]. Recent evidence indicates that FGF21 also regulates nutrient preference and reward pathways as it was reported to dramatically suppress alcohol and sweet preference by increasing neuronal activation in the paraventricular nucleus (PVN) of the hypothalamus and decreasing activity in the dopaminergic pathway in the nucleus accumbens in mice [12–14]. Interestingly, despite reducing the consumption of sweetened and alcoholic solutions, two studies have reported that FGF21 significantly increased water intake, which could be blunted by selective deletion of KLB in the brain, and suggested a potential role for FGF21 in the regulation of fluid homeostasis [13, 15].

Fluid and electrolyte balance is a tightly regulated process involving a complex network of physiologic systems and organs, including the brain and kidneys. If electrolyte concentrations (osmolality) fall outside physiologic ranges, the body rapidly responds by releasing hormones (arginine vasopressin (AVP), angiotensin II, aldosterone, etc) and modulating the autonomic nervous system in order to regulate fluid output to normalize electrolyte levels. It has been suggested that FGF21 could be involved in the hormonal and/or neuronal regulation of fluid homeostasis as FGF21 transgenic overexpression was reported to impair hypothalamic AVP expression and its circadian regulation [9, 16]. In this report, we provide a detailed characterizion of the effect of FGF21 on fluid homeostasis and explore potential mechanisms underlying these observations.

## Results

### PF-05231023 acutely increases water intake and urine output in rats

**Fig 1:**
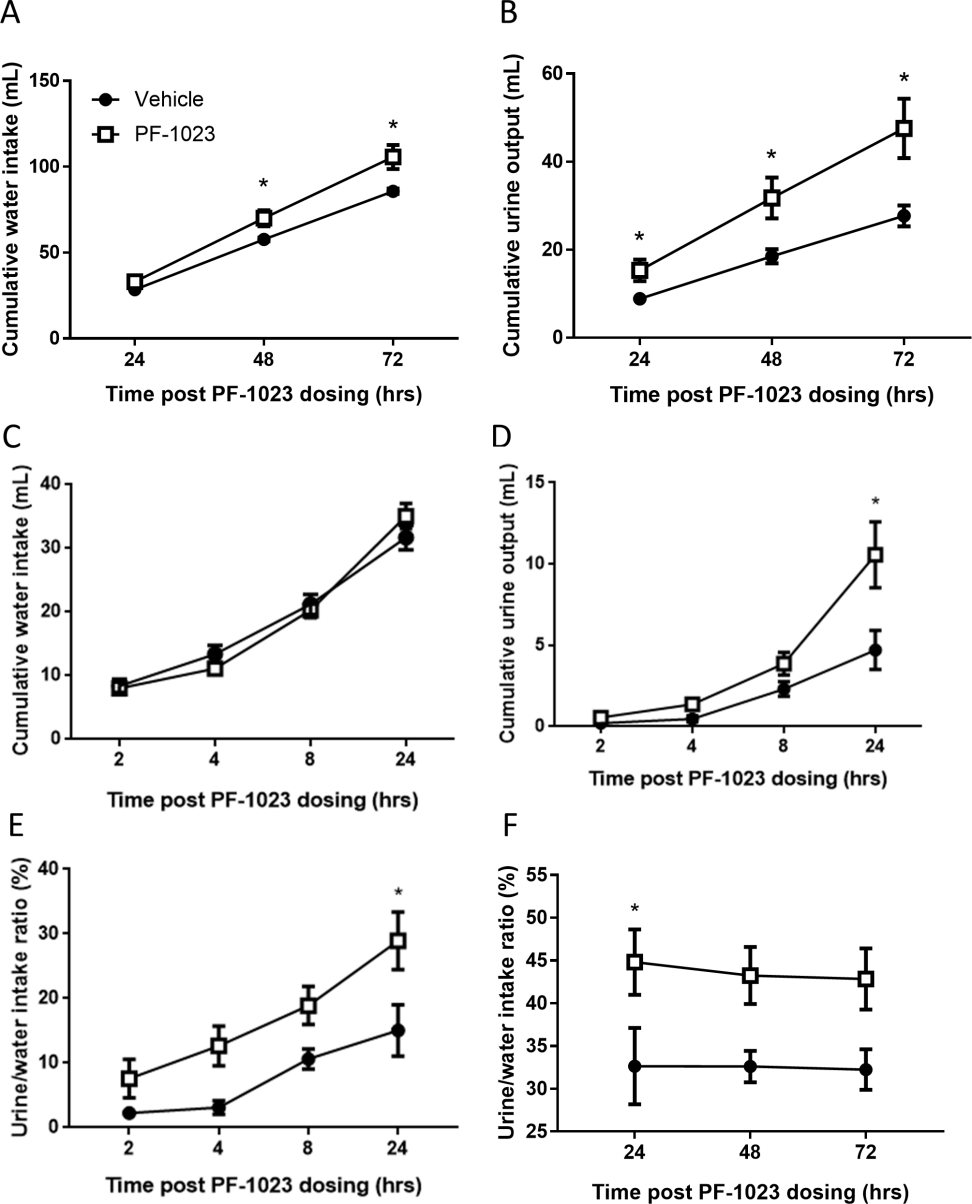
Effects of PF-05231023 on water intake and urine output in rats. Cumulative water intake (A) and urine output (B) were measured 24–72 hrs post intravenous (IV) PF-05231023 (1 mg/kg) or vehicle dosing (n = 10 per group). Cumulative water intake (C) and urine output (D) were measured 2–24 hrs post IV PF-05231023 (1 mg/kg) dosing in other PF-05231023 or vehicle treated rats (n = 10 per group). (E) Urine/water ratio was calculated using the data presented in A-D. Closed circles: vehicle-treated animals. Open squares: PF-05231023-treated animals. Data presented as mean ± SEM. Two-way repeated measure ANOVA followed by Tukey’s (A) or Sidak’s (B-F) multiple comparison tests were utilized to determine significance (S1 Table). Significant difference indicated as *p<0.05 versus vehicle control animals.

To assess the effect of FGF21 on fluid homeostasis in rats, we used PF-05231023, a long-acting analog of FGF21 that has previously been shown to improve glucose tolerance in mice and induce body weight loss in mice, non-human primates and humans [5, 17]. When rats were administered a single intravenous injection of PF-05231023, we observed a 20% increase in cumulative water intake at 48 and 72 hours post-dosing, as compared to control animals (Fig 1A). The increased water consumption was accompanied by an increase in urine output; however, interestingly, the effect on urine output appeared to preceed the increase in fluid intake (Fig 1B). By 24 hours, urine output of PF-05231023-treated rats was 40% more than control animals, and this difference was maintained throughout the study. Urine output began to increase 2 hours post drug administration (Vehicle: 0.21 mL ± 0.1 and PF-05231023: 0.55mL ± 0.19, NS) and became statistically significant at the 24 hour timepoint, whereas water consumption was unchanged during this period (Fig 1C and 1D). The difference between vehicle and PF-05231023 was further illustrated when fluid balance, calculated by expressing urine output as a percentage of water intake, was determined, and showed an increase in PF-05231023-treated animals at 24 hours and thereafter (Fig 1E and 1F).

**Fig 2:**
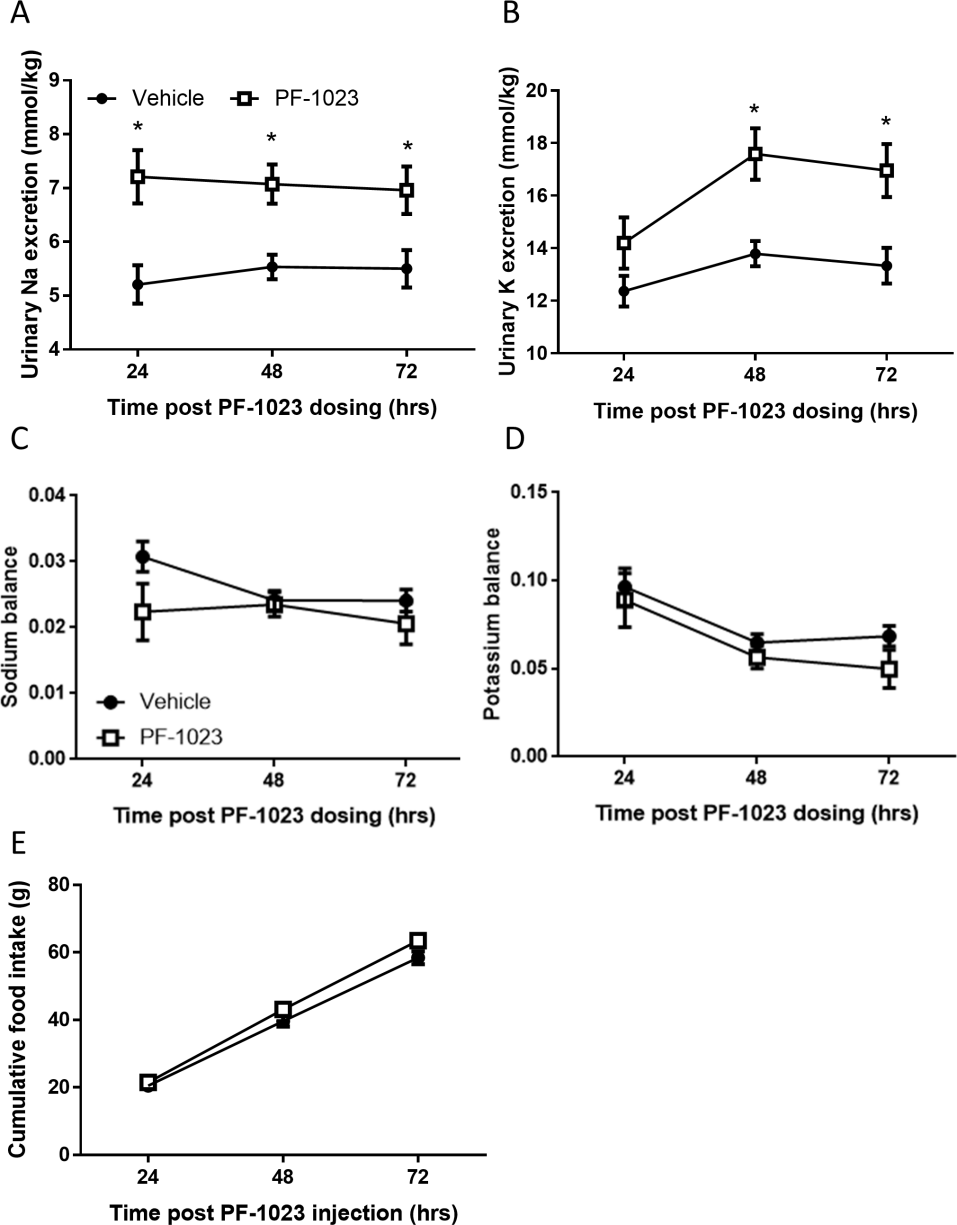
Effects of PF-05231023 on sodium/potassium balance in rats. Urinary sodium (A) and potassium (B) excretion were measured in rats treated with 1 mg/kg PF-05231023 IV (n = 10). Sodium (C) and potassium (D) balance as well as food intake (E) were also determined. Data presented as mean ± SEM. Two-way repeated measure ANOVA followed by Sidak’s multiple comparison tests were utilized to determine significance (S1 Table). Significant difference indicated as *p<0.05 versus vehicle control animals.

**Fig 3:**
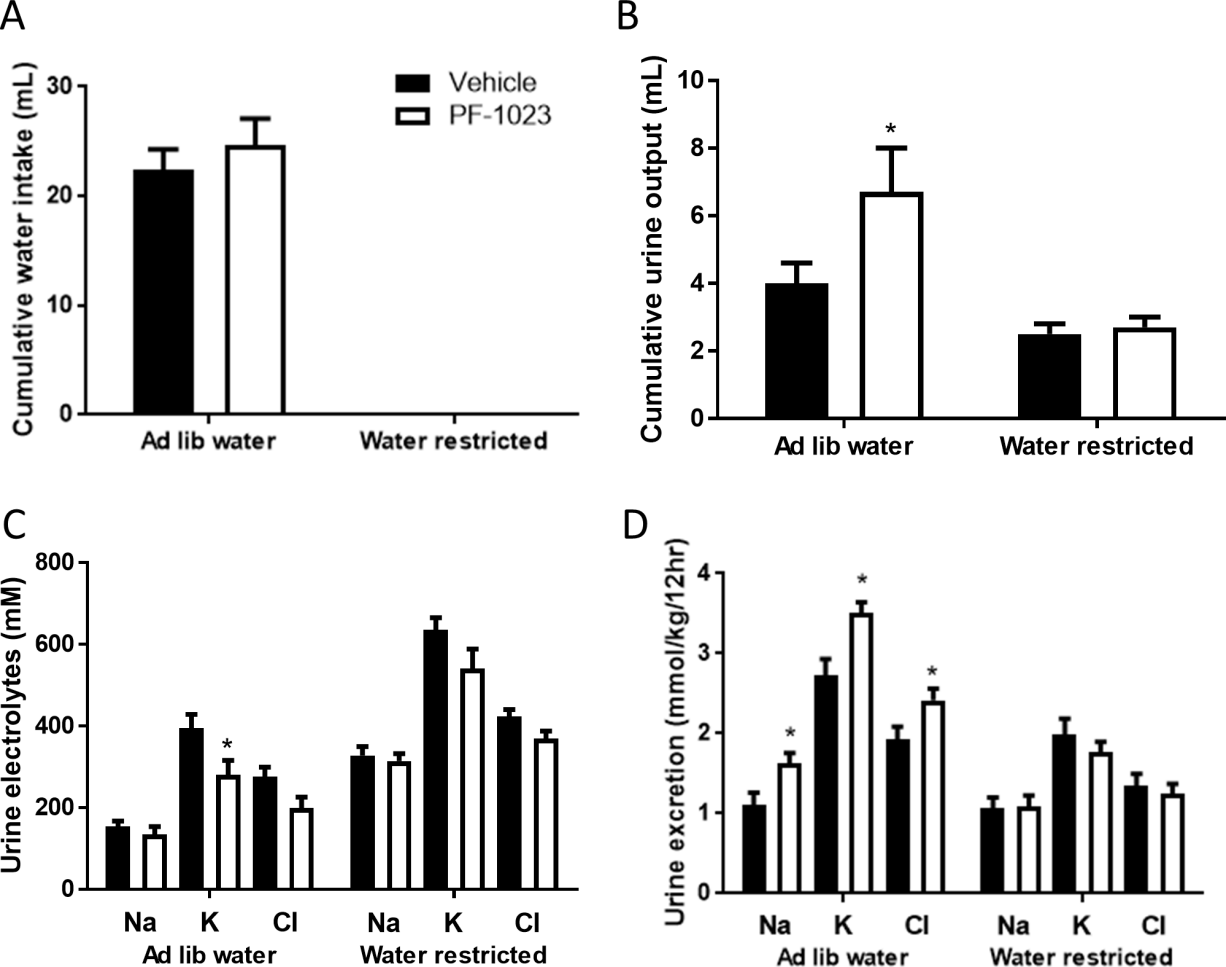
Effects of PF-05231023 during water restriction. Water intake (A), urine output (B), urine electrolyte concentrations (C) and urine electrolyte excretion (D) were measured in rats that were water restricted for 12 hours (n = 10). Water restriction was initiated immediately after dosing. Data presented as mean ± SEM. One way ANOVA followed by Tukey’s multiple comparison test was utilized to determine significance (S1 Table). Significant difference indicated as *p<0.05 versus vehicle control animals.

To understand whether PF-05231023 causes diuresis by altering kidney function, we evaluated serum and urine electrolyte levels 24 hrs post injection. Although the serum concentrations of all electrolytes evaluated were unchanged (Table 1), we found that urine concentrations of sodium, potassium and chloride were significantly decreased in PF-05231023-treated animals, consistent with a more dilute urine (Table 2). However, because PF-05231023-treated rats excreted greater volumes of urine, the total urinary excretion of sodium and potassium was significantly elevated in these animals (Fig 2A and 2B). Despite significant elevations in electrolyte excretion with PF-05231023 treatment, we observed that sodium and potassium balance was not significantly different between groups (Fig 2C–2E), potentially due to limitations in accurately measuring small differences in food intake in rodents. Indeed, we observed that PF-05231023 caused a non-significant increase in cumulative food intake (Vehicle: 58.4 g ± 2.0 and PF-05231023: 63.5 g ± 2.4, NS), but the resulting balance (electrolyte ingested minus electrolyte excretion) was not significantly different. Interestingly, the elevation in urine output and electrolyte excretion was abrogated in PF-05231023-treated animals subjected to a 12-hrs water restriction (Fig 3). Moreover, hypothalamic expression of AVP and corticotropin-releasing hormone (CRH), as well as circulating levels of corticosterone, atrial natriuretic peptide (ANP) and plasma renin activity (PRA) were unchanged by PF-05231023 treatment (S1 Fig).

**Table 1:** Serum electrolyte and creatinine concentrations in rats treated with vehicle or PF-05231023.

	Sodium (mM)	Potassium (mM)	Chloride (mM)	Creatinine (mg/dL)
**Vehicle**	147.7 ± 0.4	6.4 ± 0.4	98.7 ± 0.4	0.27 ± 0.04
**PF-05231023**	145.5 ± 0.7	7.0 ± 0.4	97.5 ± 1.1	0.25 ± 0.04

Data presented as mean ± SEM. Serum electrolytes and creatinine were measured 24hrs post-vehicle or PF-05231023 administration.

**Table 2:** Urinary electrolyte and creatinine concentrations in rats treated with vehicle or PF-05231023.

	Sodium (mM)	Potassium (mM)	Chloride (mM)	Creatinine (mg/dL)
**Vehicle**	170.7 ± 17.3	355.9 ± 40.0	268.2 ± 30.0	94.1 ± 9.9
**PF-052 31023**	113.0 ± 16.9*	185.5 ± 27.2*	151.7 ± 22.5*	48.1 ± 6.6*

Data presented as mean ± SEM with significant difference indicated as *p<0.05 versus vehicle control using Student t-test (unpaired, two tails). Urinary electrolytes and creatinine were measured 24hrs post-vehicle or PF-05231023 administration.

### PF-05231023 increases blood pressure and heart rate in rats

**Fig 4:**
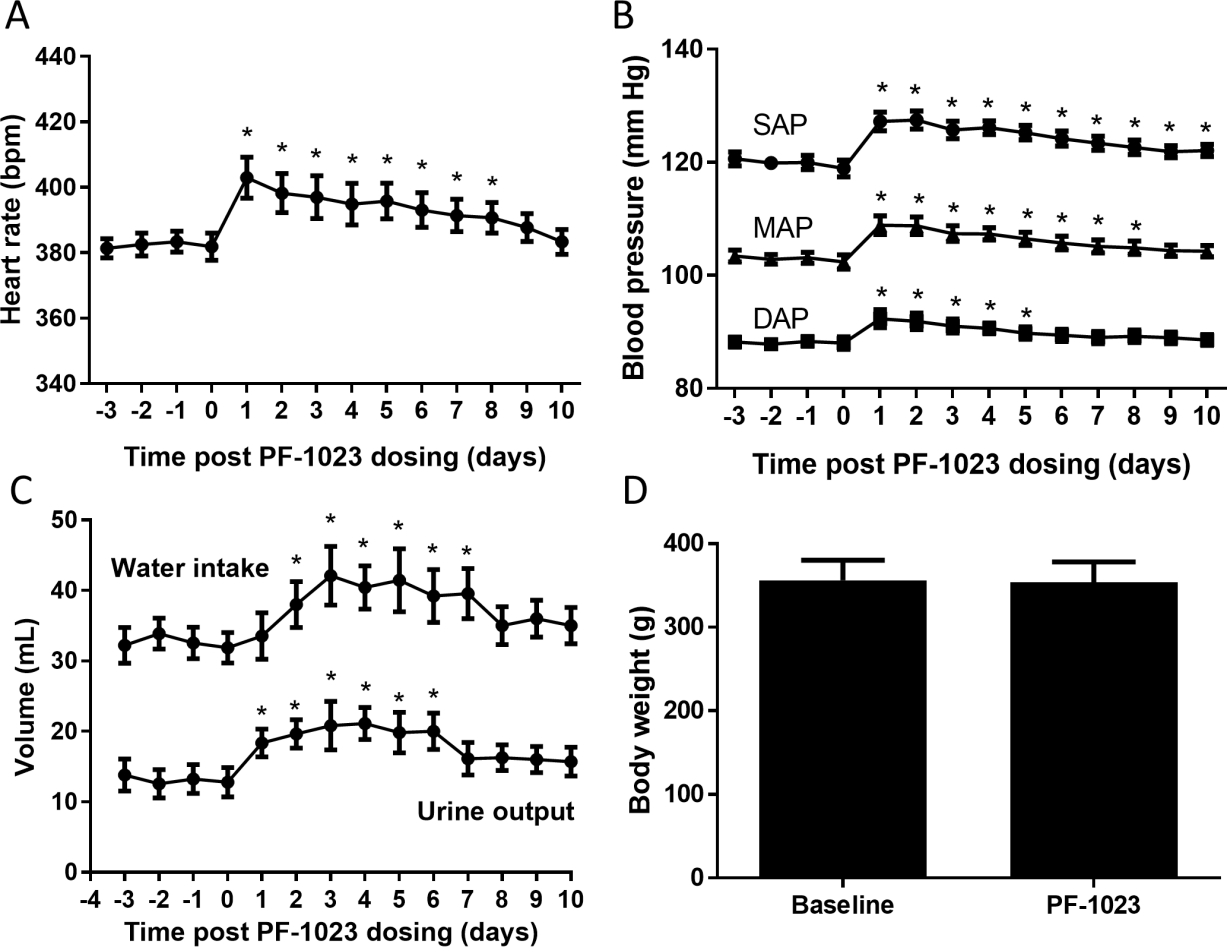
Effects of PF-05231023 on heart rate and blood pressure in rats. Heart rate, blood pressure, water intake and urine output were measured in telemeter-implanted rats that were housed in metabolic cages for a total of 17 days. (A) Heart rate and (B) blood pressure (diastolic (DAP), mean (MAP) and systolic (SAP)) as well as urine output and water intake (C) were measured 4 days before or up to 10 days after a single dose of PF-05231023 IV (10 mg/kg) injection (n = 9). Body weight (D) was measured the day before PF-05231023 administration (baseline) and 7 days post PF-05231023 administration. Data presented as mean ± SEM. Heart rate, blood pressure, water intake, and urine output were analyzed separately using PROC GLM procedure for analysis of variance (ANOVA). The model included animal and post dosing time (day) as fixed categorical factors. Comparisons to day 0 (i.e. baseline) were performed to understand PF-05231023 treatment effect. Student t-test (unpaired, two-tails) was utilized to determine significance between groups for body weight (S1 Table). A p-value less than 0.05 (i.e. p<0.05) indicates a statistically significant difference between treatments or days.

We next determined the kinetics of PF-05231023 on water intake, urine output, heart rate and blood pressure by housing telemeter-implanted rats into metabolic cages. It was previously reported that a single intravenous dose of PF-05231023 rapidly and significantly increased heart rate, systolic, mean and diastolic blood pressure 0.5–2 hours post administration [18]. Because the injection stress on these parameters is lost in less than 2 hours (S2 Fig), the next experiment was designed so that heart rate and blood pressure were recorded before (baseline) and after PF-05231023 administration so that each animal could serve as its own control. We observed that a single intravenous dose of PF-05231023 significantly increased heart rate, systolic, mean and diastolic blood pressure and the effects gradually decreased over time but remained significantly elevated for 5 days (diastolic blood pressure), 8 days (heart rate) or 10 days (mean and systolic blood pressure) and were independent of body weight change (Fig 4). Similar to observations in non-telemeterized animals, urine output and water intake were significantly elevated by 24 hrs and 48 hrs post-dosing, respectively, and the effects were sustained for up to 6 days post-treatment with urine output declining first, followed by water intake (Fig 4C).

### Effect of PF-05231023 on baroreflex

**Fig 5:**
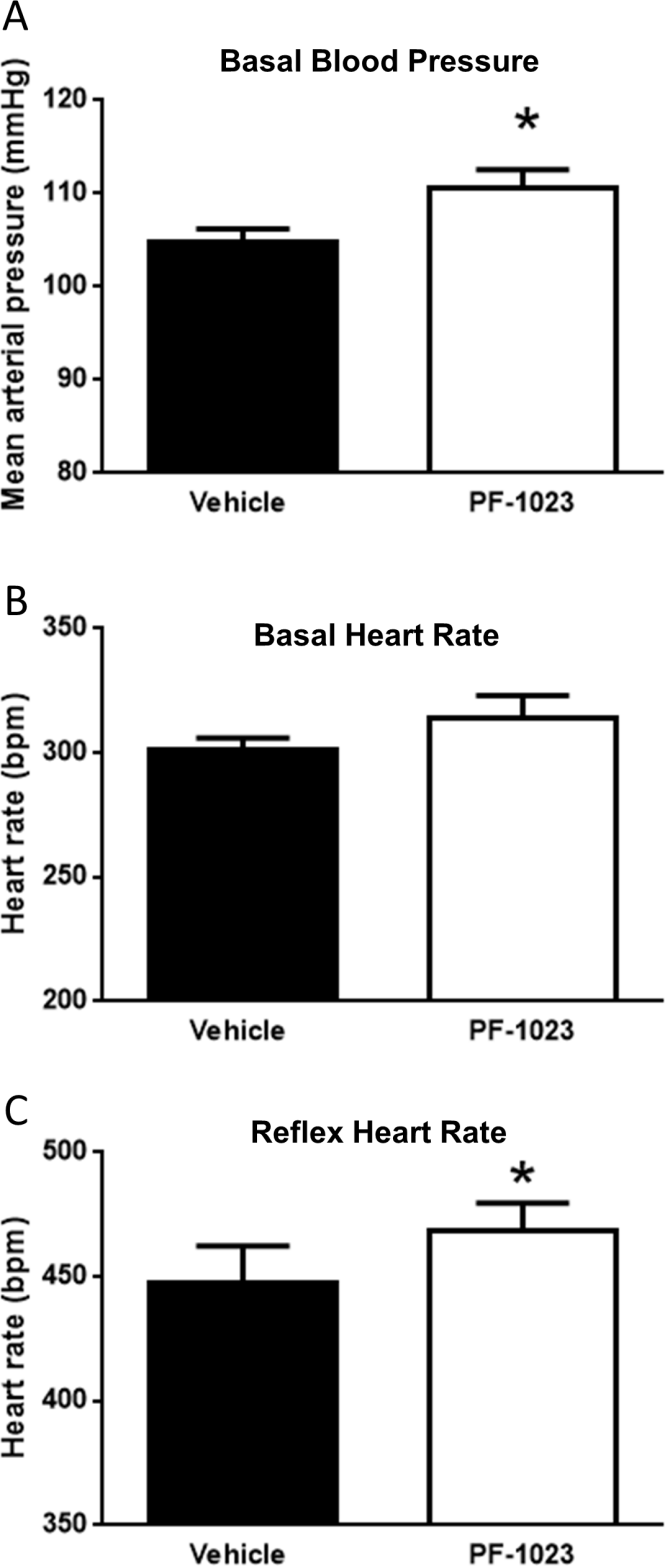
Effects of PF-05231023 on cardiac baroreflex response. Baseline mean arterial pressure (A), heart rate (B), and cardiac baroreflex response to SNP-induced decrease in arterial pressure from baseline to 70 mmHg (C) were measured in the same conscious rats (n = 9) ~ 60 min after either vehicle (control) or PF-05231023 (20 mg/kg) treatment. Paired testing was seperated by 2 days. Data presented as mean ± SEM. Student t-test (paired) was utilized to determine significance. Significant difference indicated as *p<0.05 versus vehicle period.

Because PF-05231023 rapidly elevates blood pressure and heart rate after dosing, we hypothesized that PF-05231023 is acting through the sympathetic nervous system to mediate its effects. To assess the potential effect of PF-05231023 on the sympathetic nervous system, we performed cardiac baroreflex tests by decreasing arterial pressure using the systemic vasodilator sodium nitroprusside [19, 20]. The maximal dose of sodium nitroprusside reduced arterial pressure to 70 mmHg. Sixty minutes after the initial dosing of PF-05231023 and prior to reflex testing, we detected a significant increase in baseline mean arterial pressure by 6 mmHg and a non-significant increase in baseline heart rate by 13 bpm (Fig 5A and 5B). PF-05231023 treatment significantly increased baroreflex-induced cardiac reflex tachycardia compared with vehicle, suggesting that blood pressure response is mediated in part by increased synpathetic activity (Fig 5C).

**Fig 6:**
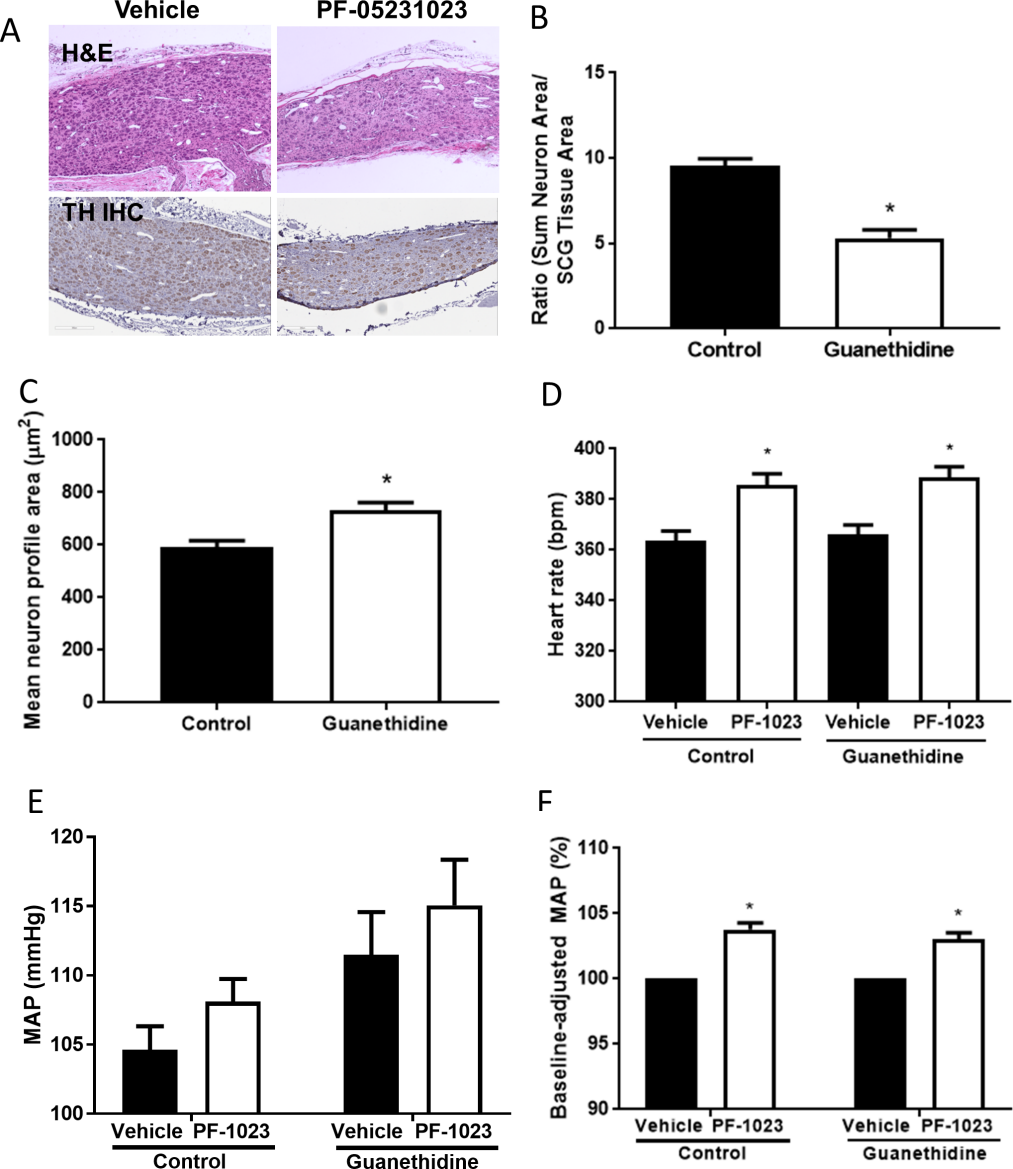
Effects of PF-05231023 on heart rate and blood pressure in guanethidine-treated rats. (A) Representative sections of hematoxylin & eosin (H&E) and tyrosine hydroxylase (TH)-immunostained superior cervical ganglia (SCG) from animals that received PF-05231023 (10 mg/kg IV) 4 months post vehicle (n = 9) or guanethidine (n = 12) treatment. Total neuron number (B) and neuronal volume (C) in SCG of control and guanethidine-treated rats. Heart rate (D), blood pressure (E) and baseline-adjusted blood pressure (F) measured during the baseline vehicle or PF-05231023 periods in control or guanethidine-treated animals. Data presented as mean ± SEM. Student t-test was utilized to determine significance for panels B and C. Two-way ANOVA followed by Sidak’s multiple comparison tests were utilized to determine significance (D-F). Significant difference indicated as *p<0.05 versus control group or pre-PF-05231023 (vehicle) period (S1 Table).

To further elucidate the involvement of FGF21-driven sympathetic activation on blood pressure and heart rate, we used guanethidine to permanently deplete sympathetic post-ganglionic neurons as previously reported [20, 21]. Guanethidine treatment resulted in a 44% reduction in the number of superior cervical ganglionic (SCG) neurons (Fig 6A and 6B). Although fewer neurons were present in the SCG of guanethidine-treated animals, we observed that the remaining neurons were larger in comparison to the neurons found in the control group (Fig 6C). In contrast to the published model [21], we observed some residual effects of guanethidine on baseline blood pressure 28 days post-treatment (Fig 6E). The baseline heart rate was not significantly changed in guanethidine-treated animals (Fig 6D) while baseline MAP was elevated by approximately 7 mmHg (control: 104.5 ± 0.9 bpm, guanethidine: 111.3 ± 1.3 bpm, p = 0.06, Student t-test). Similar to our related findings (Fig 4), PF-05231023 treatment increased heart rate and baseline-adjusted mean arterial blood pressure in control rats but the FGF21 analog similarly increased these parameters in guanethidine-treated animals (Fig 6D–6F).

## Discussion

To assess the effects of FGF21 on fluid homeostasis and blood pressure in our studies, we used a long-acting analog, PF-05231023, that has previously been shown to improve glucose tolerance in mice and induce body weight loss in mice, non-human primates and humans [5, 17]. In contrast to the reported weight loss effects observed in several species, we did not detect any significant change in food intake or body weight in our studies (Figs 2E and 4D, respectively). This result was not completely unexpected as it has been previously reported that native FGF21 as well as PF-05231023 do not induce weight loss in diet-induced obese (DIO) rats and Zucker diabetic fatty (ZDF) rats, despite significantly improving glucose metabolism [22, 23]. The molecular mechanism responsible for the discrepancies observed accross species has not been completely elucidated but could implicate inter-species differences in neurocircuitry that controls appetite and energy expenditure.

It has been previously reported that a subcutanous infusion of human recombinant FGF21 can increase water intake without affecting caloric intake in mice [15]. Moreover, transgenic overexpression of FGF21 in mice resulted in an approximately 9-fold increase in water intake, which was completely abrogated in brain-specific KLB knockout animals [13]. In line with these earlier observations, we showed that a single dose of PF-05231023 increased water intake in rats, without significantly impacting body weight or food intake. Water intake remained elevated for 6 days after single administration of PF-05231023 and the effect was sustained for up to 14 days when the FGF21 analog was administered every 3 days [24].

Small increases in plasma osmolality result in stimulation of thirst in mammals [25]. Indeed, injection of hypertonic salt solutions causes rats to drink more water than animals receiving isotonic saline [26]. Moreover, hormones, including renin and angiotensin II, are highly effective at stimulating water intake in sated rats [25]. In our studies, we did not detect any change in serum electrolyte levels with FGF21 nor did we observe changes in hormonal factors known to be involved in the physiological regulation of thirst. In healthy individuals, the body typically responds to an elevation in fluid intake by causing mild diuresis in order to maintain adequate fluid balance [27, 28]. As expected from the increased fluid intake observed in our studies, we detected a significant elevation in urine output post FGF21 administration. Interestingly, a significant increase in urine volume was detected 24 hours after administration of PF-05231023, prior to measurable increase in water intake. These results were replicated in a separate set of experiments using telemeter-implanted rats. The kinetics of diuresis and increased water intake was unexpected and suggest that FGF21-induced water intake is secondary to diuresis in rats.

The rate of urine output is tightly regulated by varying filtration in different parts of the nephron, where variable amounts of water and salt are removed under the control of hormones, including AVP and aldosterone. We questioned whether the effect of FGF21 on diuresis could be mediated through a direct renal action. FGF21 is known to mediate its effects by binding KLB through its C-terminus portion while the N-terminus of FGF21 displays affinity to several FGFRs, including FGFR1c. It is widely accepted that a close relative to KLB, klotho, is highly expressed in kidneys but the presence of KLB in this organ is less characterized. We detected high levels of FGFR1c and klotho in the cortex and medulla of rat kidneys but the expression of KLB was several orders of magnitude lower (S3 Fig). These data suggest that although KLB is detectable at low levels in the kidney, it is unlikely that FGF21 is regulating diuresis by a direct KLB/FGFR1c renal mechanism. Even though the observed changes in urinary electrolyte excretion by PF-15231023-treated rats suggest changes in the control of urine output, we did not detect any change in hormonal factors known to be involved in the physiological regulation of diuresis (plasma renin activity, CRH, corticosterone, AVP) after PF-05231023 treatment. Furthermore, the increased excretion of electrolytes (Na, K, Cl) caused by PF-05231023 administration was completely reversed upon water restriction suggesting that the kidneys were normally responding to hormonal cues to adjust electrolytes and water homeostasis. The fact that key components of the hormonal pathways regulating fluid balance were unchanged, combined with a normal response to water deprivation and low level of expression of KLB in the cortex and medulla of the kidney suggested that PF-05231023 is regulating diuresis through an alternate pathway.

The long term regulation of blood pressure is linked to the ability of the kidneys to modulate tubular sodium reabsorption, which in turn increases or decreases water excretion and keeps the extracellular fluid volume and blood pressure within normal ranges. Elevations of renal perfusion pressure under experimental conditions have been shown to increase urine flow and sodium excretion in normotensive animals [29]. This mechanism, called pressure natriuresis, occurs as a compensatory mechanism and acts to maintain blood pressure within physiological ranges. The increase in sodium excretion and urine output observed with FGF21 treatment in our studies, in the absence of changes in renal regulatory hormones, led us to postulate that natriuresis and diuresis were secondary to FGF21 induced elevation in blood pressure. Emerging studies have started investigating the role of FGF21 in blood pressure regulation but the relationship between both factors is not fully understood [18, 30]. FGF21 is generally believed to decrease blood pressure through its dramatic improvements in lipid profiles as accumulating evidence suggests that obesity and dyslipidemia are key risk factors in the development of hypertension [31–33]. However, epidemiological studies do not agree with this hypothesis, as circulating levels of FGF21 have been positively correlated with systolic blood pressure in several adult populations [34–38]. Consistent with epidemiological studies, a recent report has shown that PF-05231023 increases blood pressure in rats and humans [18]. These data are also in agreement with a recent study indicating that intravenous administration of recombinant human FGF21 acutely increases mean arterial blood pressure, although not significantly, in anesthetized lean and high-fructose drinking rats [30]. We demonstrated here that a single dose of our FGF21analog indeed increased blood pressure in lean rats and the effect was observed rapidly after its administration and sustained for several days. We believe that the sustained effect of PF-05231023 on blood pressure is likely due to the pharmacokinetic properties of the molecule as the elimination half-life of PF-05231023 was reported to be 97.3hr, 22.7hr and 6.3hr for the intact, N-terminal portion or C-terminal portion of PF-05231023 [39] and the actual exposure was several fold above the in vitro potency (IC_50_ of 0.5 nM) for multiple days. Our study, combined with the recent report demonstrating that PF-05231023 rapidly elevates blood pressure in rats and humans [18], suggests that PF-05231023 causes a rapid elevation in heart rate/blood pressure, followed by an increase urine excretion and then water intake. Based on the kinetics of these events, we thus propose that FGF21 increased urine output as a compensatory mechanism to alleviate the elevation in blood pressure.

The mechanism by which FGF21 increases blood pressure is not fully understood but the rapid onset of action led us to hypothesize that PF-05231023 could be acting through the autonomic nervous system. The brain is part of a complex regulatory network receiving input from mechanical (barosensory) and chemical (chemosensory) sensors located peripherally and sending rapid and precise responses through sympathetic and parasympathetic effectors. FGF21 has previously been shown to stimulate brown adipose tissue sympathetic activity by centrally activating the KLB/FGFR pathway [9]. More recently, FGF21 was also proposed to mediate some of its effects through activation of the autonomic system as recombinant human FGF21 acutely increased the baroreflex sensitivity in normal as well as high fructose drinking rats [30]. Consistent with these studies, our data showing reflex tachycardia also suggested sympathetic activation by FGF21.

It has been previously shown that guanethidine treatment produces profound and significant permanent depletion of sympathetic postganglionic neurons in experimental animals and the morphometric analysis of postganglionic neurons in the current study is in agreement with published literature [21, 40–45]. However, PF-05231023 was still capable of elevating blood pressure and heart rate in rats in which approximately 40% of the sympathetic post-ganglionic neurons were ablated. The remaining sympathetic neurons in guanethidine-treated animals were larger than control neurons which suggests a neuroplastic adaptation to compensate for the neuronal loss. Thus, we believe based on our reflex tachycardia response that FGF21 indeed activates the sympathetic nervous system but that the remaining larger neurons in guanethidine-treated rats were sufficient to drive the FGF21 effects on blood pressure and heart rate.

In summary, our data suggest that FGF21-induced water intake is in fact secondary to diuresis, which we believe is a compensatory mechanism engaged to alleviate the hypertension caused by our FGF21 analog. Although our data suggest that sympathetic activation by FGF21 might be responsible for the increased blood pressure observed in this study, additional work is required to fully understand the contribution of the sympathetic nervous system in the regulation of blood pressure with FGF21.

## Materials and methods

### Reagents

PF-05231023 was produced as previously described [39]. Guanethidine was purchased from Apexbio technology LLC.

### Ethics statement

All procedures performed on animals in this study were in accordance with the humane guidelines for ethical and sensitive care by the Institutional Animal Care and Use Committee (IACUC) of the US National Institutes of Health. All experimental work conducted for this study was approved by Pfizer IACUC. Pfizer animal care facilities that supported this work are fully accredited by AAALAC International.

### Animal studies

Animals were housed in static microisolator cages or in Innocage^®^ cages (Innovive, San Diego, CA) on ventilated racks on Alpha-Dri^®^ (Shepherd Specialty Papers Watertown, TN) bedding, or in metabolic cages without bedding, and maintained at 21 ± 1°C, 40–70% RH. Rats were fed Lab Diet #5053 chow (Purina, St-Louis, MO) and water *ad libitum*, except for water restriction studies. Studies were performed in reverse-light cycle (12:12-hour dark:light), unless otherwise indicated, to ensure endpoints were collected during the animals´ active phase of the day. Animals were acclimated to the reverse light cycle for at least 15 days before study initiation.

### Effects of PF-05231023 on water intake and urine output

Eight-to-nine-week-old male Wistar Han rats [Crl:WI (HAN)] (Charles River, Wilmington, MA) were housed 2 per cage and acclimated as described above. On the day of the experiment, animals were weighed and assigned to treatment group so that each group had a similar range of body weights (n = 10/group). Animals then received a single dose of vehicle (30mM Lactate, 9% Trehalose, 0.05mg/ml EDTA, 0.1MG/ml L- Methionine, 0.5mg/ml Tween) or PF-05231023 (1 mg/kg) intravenously through the tail vein one hour before the onset of the dark phase, and housed (1/cage) in metabolic cages with *ad libitum* access to water and food. A dose of 1 mg/kg was chosen for these studies because it provided exposure above in vitro IC_50_ for several hours [39] and had been previously demonstrated to sub-maximally improve glucose tolerance in mice without causing weight loss [17]. Water intake and urine output were measured 2, 4, 8, 24, 48 and 72 hrs post-dosing. Food intake was measured 24, 48 and 72 hrs post-dosing. Twenty-four hour post-dosing, a subset of animals were euthanized with CO_2_ and blood, hypothalamus and kidney cortex and medulla were collected for further assessment (n = 3–9 for gene expression and n = 10 for blood analysis).

### Effects of PF-05231023 on electrolytes and circulating factors

Sodium and potassium balance was calculated as the difference between the amount of sodium/potassium ingested and excreted in urine. For water restriction studies, animals were weighed and assigned to treatment group so that each group had a similar range of body weights (n = 20/group), received a single dose of vehicle (30mM Lactate, 9% Trehalose, 0.05mg/ml EDTA, 0.1MG/ml L- Methionine, 0.5mg/ml Tween) or PF-05231023 (1 mg/kg) intravenously through the tail vein one hour before the onset of the dark phase, and housed (1/cage) in metabolic cages. Half the animals were not allowed access to water for a 12-hour period before they were euthanized by CO_2_ asphyxiation. For corticosterone and plasma renin activity measurements, 8-week old male Wistar Han rats [Crl:WI(HAN)] (Charles River, Wilmington, MA) with jugular vein catheters were received from the vendor and allowed to acclimate for 12 days (single housed) before the beginning of the experiment. Rats received a single dose of vehicle (same as above) or PF-05231023 (1 mg/kg) intravenously through the tail vein one hour before the onset of the dark phase and blood was collected via jugular catheter 24 hrs later in conscious unrestrained animals to minimize the effect of stress on circulating endpoints. For plasma renin activity, blood was collected via jugular catheter in EDTA tubes at room temperature (RT), centrifuged at RT and plasma was frozen within an hour of blood collection. Serum was used for corticosterone measurements.

### Surgical implantation of telemetry devices and indwelling venous catheters

Aseptic technique was strictly adhered to for all surgical procedures as well as during the experimental preparations and the routine flushing of the indwelling catheters. Animals were anesthetized using 2–3% isoflurane in O_2_ in a clear plastic box. During surgery, anesthesia was maintained with 1–3% isoflurane administered via a nosecone. The surgical site was clipped free of hair and prepared for aseptic surgery using alternating wipes of appropriate surgical scrub, 70% ethanol, and sterile saline. Ophthalmic ointment was instilled in the animal's eyes to prevent corneal drying. Anesthetic depth, respiratory rate and pattern were frequently monitored. Depth of anesthesia was assessed by loss of movement, loss of blink reflex, and lack of response to stimulation (such as toe pinch). Toe pinch was monitored at 10–15 minute intervals through the surgery. Animals were also provided with supplemental heat to maintain their body temperatures.

An abdominal midline incision was made extending to slightly above the xiphoid cartilage. Abdominal wall and viscera were covered with saline moistened gauze. Intestines were retracted to expose and isolate the descending aorta. A circular purse string suture was placed in the aorta tunica using a non-absorbable suture. Two clamps were used to occlude the aorta, one on the aorta near the renal artery and the other at the common iliac bifurcation. The aorta was pierced in the center of purse string using a curved needle and the catheter was guided into the vessel retrograde with the tip resting distal to the renal artery. The purse string suture was quickly tightened down around the catheter to seal the juncture. Clamps were removed to allow the timely reperfusion of distal body parts. The telemetry device with suture ribs was then fixed to the abdominal wall with non-absorbable suture. The peritoneal cavity was then irrigated with warm, sterile saline and the intestines put back in place. Incisions and subcuticular layers were closed with absorbable suture. For baroreflex testing, rats were surgically implanted with indwelling femoral venous catheters few weeks after the telemetry surgery for the administration of vasoactive drugs. The catheter was tunneled under the skin and exposed at the dorsal surface of the neck as previously described. Animals received thermal support in the immediate post-operative phase and returned to clean cages once appropriately recovered. Buprenorphine in combination with meloxicam or buprenorphine and carprofen were provided for a minimum coverage of 72 hours post-surgery. Rats were monitored postoperatively for well-being, pain, and incisional healing twice daily for 7 days.

### Effects of PF-05231023 on blood pressure, heart rate, water intake and urine output

Twelve-week-old male Wistar Han rats [Crl:WI (HAN)] (Charles River, Wilmington, MA) were acclimated to our facility under standard 12:12 light dark cycles for 14 days before they were implanted with DSI PA-C40 blood pressure (BP) and activity telemeters (Data Science International, St. Paul, MN) as described above. Approximately 2 weeks post-surgery, animals (n = 9) were transfered to metabolic cages fitted with telemetry receivers and baseline blood pressure, heart rate, water intake, and urine output captured. One week later, animals received a single IV dose of PF-05231023 (10 mg/kg) through the tail vein and data was collected for an additional 10 days. Studies performed to determine the cardiovascular safety pharmacology of PF-05231023 in rats showed that the magnitude and duration of effect on blood pressure and heart rate increased with dose [18]. Although significant, the effects of PF-05231023 at 1 mg/kg were relatively mild so we picked the highest dose tested of 10 mg/kg to maximize our chances of seeing significant changes in this study. Throughout the experiment, water intake and urine output were collected daily while telemetry data were collected continuously at 500 Hz sampling rate from individual animals using DSI Dataquest A.R.T. (version 4.36) system. Heart rate (HR) was derived the blood pressure waveform. Blood pressure and heart rate measurements were logged continuously as 1-minute means. Daily (24-hour) means of blood pressure and heart rate measurements were obtained by binning 1-minute means apart from the dosing time.

### Cardiac baroreflex testing

Adult male Wistar Han rats (n = 9) were implanted with telemetry devices (TL11M2-C50-PXT/PT or HD-S10/11, Data Science International, St. Paul, MN) and indwelling venous catheters as described above and maintained in regular light:dark cycles (12hr light/dark). On the day of the experiment (few days after venous catheter surgery), telemetry devices were activated and cardiovascular measurements were recorded at a frequency of 500Hz. The indwelling venous catheter was connected to an 18″–24″ piece of polyethylene tubing for the administration of sodium nitroprusside (SNP) for vasodilation as previously described [20]. Rats were jacketed and connected to a tether to protect the polyethylene tubing from becoming damaged and given at least 60 min to acclimate to the tether and to allow blood pressure and heart rate to stabilize from handling. Next, all rats (n = 9) were administered vehicle (saline 2 ml/kg) via venous catheter followed by a 60 min recovery period and then baroreflex testing. Two days later the same rats were administered PF-05231023 at a dose of 20 mg/kg (10 mg/ml, i.v. bolus via venous catheter) followed by a 60 min recovery period and then baroreflex testing. In both instances, after PF-05231023 (or vehicle) treatment and the stabilization period, baseline blood pressure and heart rate were recorded and baroreflex testing was performed. To produce a cardiac baroreceptor-induced tachycardia we induced changes in arterial pressure by administering SNP (4–10 μg/kg, 100 μg/ml). The SNP infusion rate was slowly increased or decreased to maintain the rat at 70 mmHg over 1–2 min. When a stable blood pressure of ~70 mmHg was met (typically 1 min after the start of the infusion) the corresponding heart rate was recorded for ~15–20 seconds.

### Effects of guanethidine on blood pressure and heart rate

Nine-to-ten-week-old male Wistar Han rats [Crl:WI (HAN)] (Charles River, Wilmington, MA) were acclimated for 1 week (single housed) in Innocage^®^ cages as described above before they received daily intraperitoneal dose (IP) of vehicle (n = 9) or guanethidine (100 mg/kg, n = 12) for 11 days. Rats had free access to food and water during the experiment. Because guanethidine has been shown to decrease food and water consumption at the dose used in this study [21], rats were weighed daily and monitored closely for clinical signs of dehydration, malaise, and general discomfort. As a precaution, rats were provided DietGel Boost (Clear H2O, Portland, ME) as a dietary supplement. Ten to 17 days post vehicle or guanethidine treatment, rats were implanted with DSI telemeters (PA-C40) and allowed to recover from surgery 14–21 days before they were transferred to metabolic cages fitted with telemetry receivers. Telemetry device implantation surgery and data acquisition were performed as described above. Blood pressure was recorded continuously and water intake and urine output were captured daily. After 4 days of baseline recording, all animals received a single dose of vehicle IV through the tail vein and the recording continued for 4 days. Then animals received a single dose of PF-05231023 (10 mg/kg) IV through the tail vein and blood pressure and heart rate were recorded for an additional 3 days. Baseline-adjusted MAP was calculated as a percentage of the last day of the vehicle period. Four months after the study was completed, animals were transcardialy perfused with 4% methanol-free formaldehyde and the superior cervical ganglia (SCG) were harvested, embedded in paraffin, sectioned and stained with Hematoxylin & Eosin (H&E) or immunostained for tyrosine hydroxylase (TH). Rat SCG sections stained with TH were scanned on the Leica/Aperio AT2 whole slide digital scanner using the 40X magnification setting and image analysis performed using HALO software. Image analysis consisted in assessing neuronal count and neuron size in one central plane of section per animal. To identify neurons consistently between samples, only neurons with TH-positive cytoplasm containing at least one distinct nucleolus within a nucleus were manually outlined.

### Clinical chemistry and circulating factor measurements

Electrolytes were measured using an Advia clinical analyzer. Corticosterone levels were measured by LC/MS/MS, plasma renin activity and pro-atrial natriuretic peptide were measured by ELISA (ALPCO Diagnostics Renin Activity ELISA, proANP (1–98), Biomedica Gruppe).

### Gene expression

Kidney cortex (1 pole) and half the medulla as well as hypothalamus collected 24 hours post-treatment were used to determine AVP, CRH, FGFR1c, KLB and klotho gene expression. RNA was extracted from cells using the Qiagen RNeasy 96 kit (Qiagen 74181) including a DNaseI digestion step (Qiagen 79254). Reverse transcription was performed using TaqMan Reverse Transcription Reagents (Life Technologies N8080234) and quantitative real-time PCR was performed with Taqman Universal PCR Master Mix (Life Technologies 4304437) using gene specific probes from Life Technologies. Gene expression of each biological sample (n = 3) was performed in triplicate.

### Statistical analysis

Data are presented as mean ± SEM. Statistical analysis was performed by either Student t-test or ANOVA followed by *post hoc* analysis using Graphpad Prism software or by using a general linear model (GLM procedure) using SAS v9.4 (SAS, Cary, NC). The details of the tests utilized can be found in the legends to figures and S1 Table. The criteria for statistical significance was set as p<0.05; p values shown refer to the post-hoc analysis.

## Supporting information

S1 Fig Effects of PF-05231023 on hypothalamic gene expression and circulating hormones. Hypothalamic (A) arginine vasopressine (AVP) and (B) corticotropin-releasing hormone (CRH) gene expression and circulating (C) corticosterone, (D) atrial natriuretic peptide and (E) plasma renin activity measured 24 hours post vehicle or PF-05231023 (1 mg/kg) IV injection (n = 3–10). Data presented as mean ± SEM. Student t-test (unpaired, two tails) was utilized to determine significance.
(TIF)

S2 Fig Heart rate and blood pressure in vehicle-treated rats. Heart rate (A) and mean arterial blood pressure (B) were measured immediately before and after intravenous administration of vehicle in telemeter-implanted rats that were housed in metabolic cages. Heart rate and blood pressure were analyzed separately using PROC GLM procedure for analysis of variance (ANOVA). The model included animal and post dosing time (min) as fixed categorical factors. Comparisons to 0 min (i.e. baseline) were performed to understand the vehicle effect.
(TIF)

S3 Fig Gene expression in kidney cortex and medulla. Gene expression of FGFR1c (A), KLB (B) and klotho (C) was assessed in the kidney cortex and medulla of vehicle-treated rats (n = 3). Data presented as mean ± SEM with Ct values shown for each dataset. Student t-test (unpaired, two tails) was utilized to determine significance.
(TIF)

S1 Table Statistical information for results presented. (DOCX)

S2 Table Raw data supporting manuscript. (XLSX)
