# Supplementary figures and images for: FGF21 increases water intake, urine output and blood pressure in rats
Source: PLoS One. 2018 Aug 14;13(8):e0202182. doi: 10.1371/journal.pone.0202182 (PMC6091943; doi:10.1371/journal.pone.0202182)

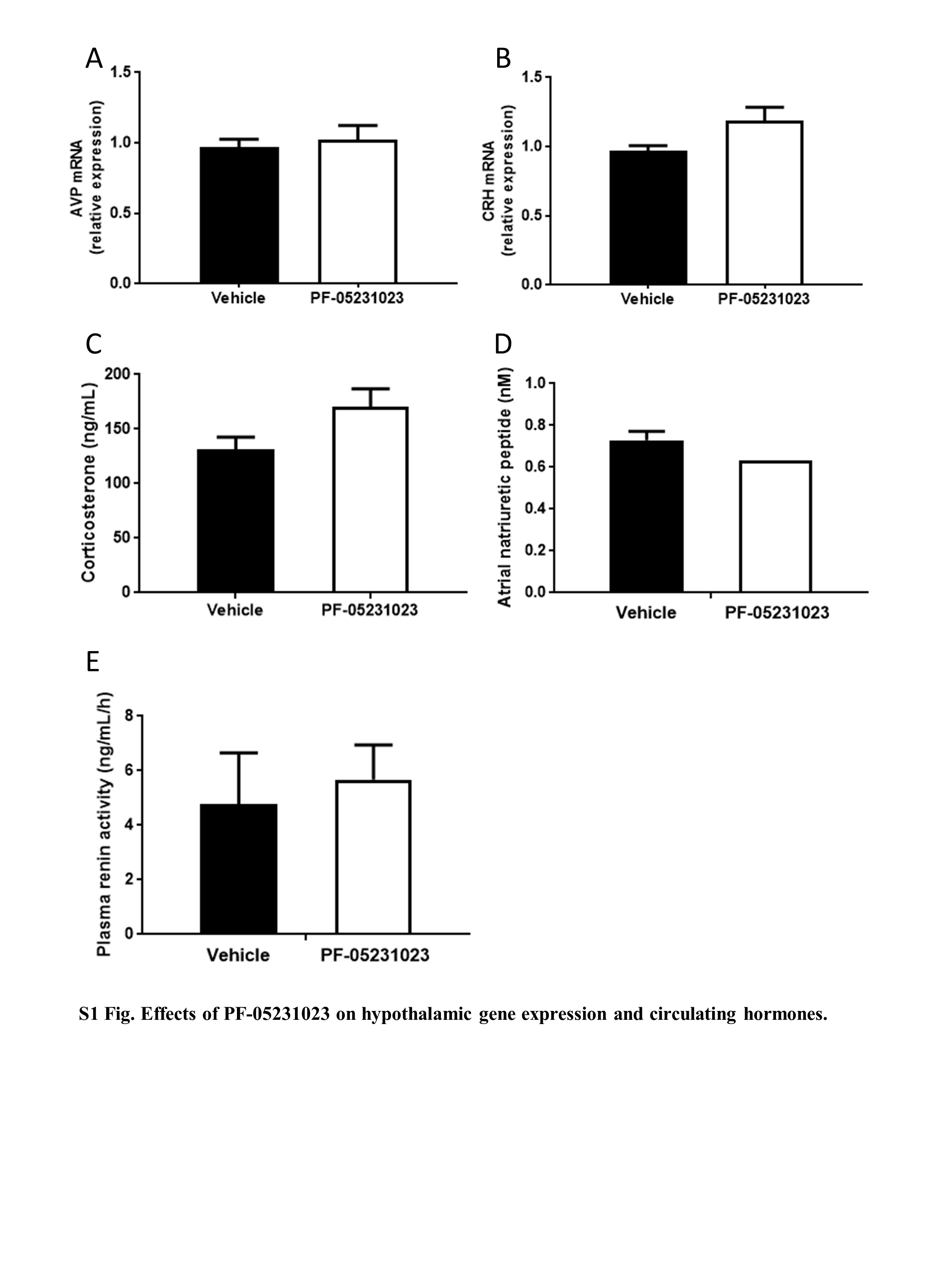

Supplement: S1 Fig — Hypothalamic (A) arginine vasopressine (AVP) and (B) corticotropin-releasing hormone (CRH) gene expression and circulating (C) corticosterone, (D) atrial natriuretic peptide and (E) plasma renin activity measured 24 hours post vehicle or PF-05231023 (1 mg/kg) IV injection (n = 3–10). Data presented as mean ± SEM. Student t-test (unpaired, two tails) was utilized to determine significance. (TIF) [file pone.0202182.s001.tif]

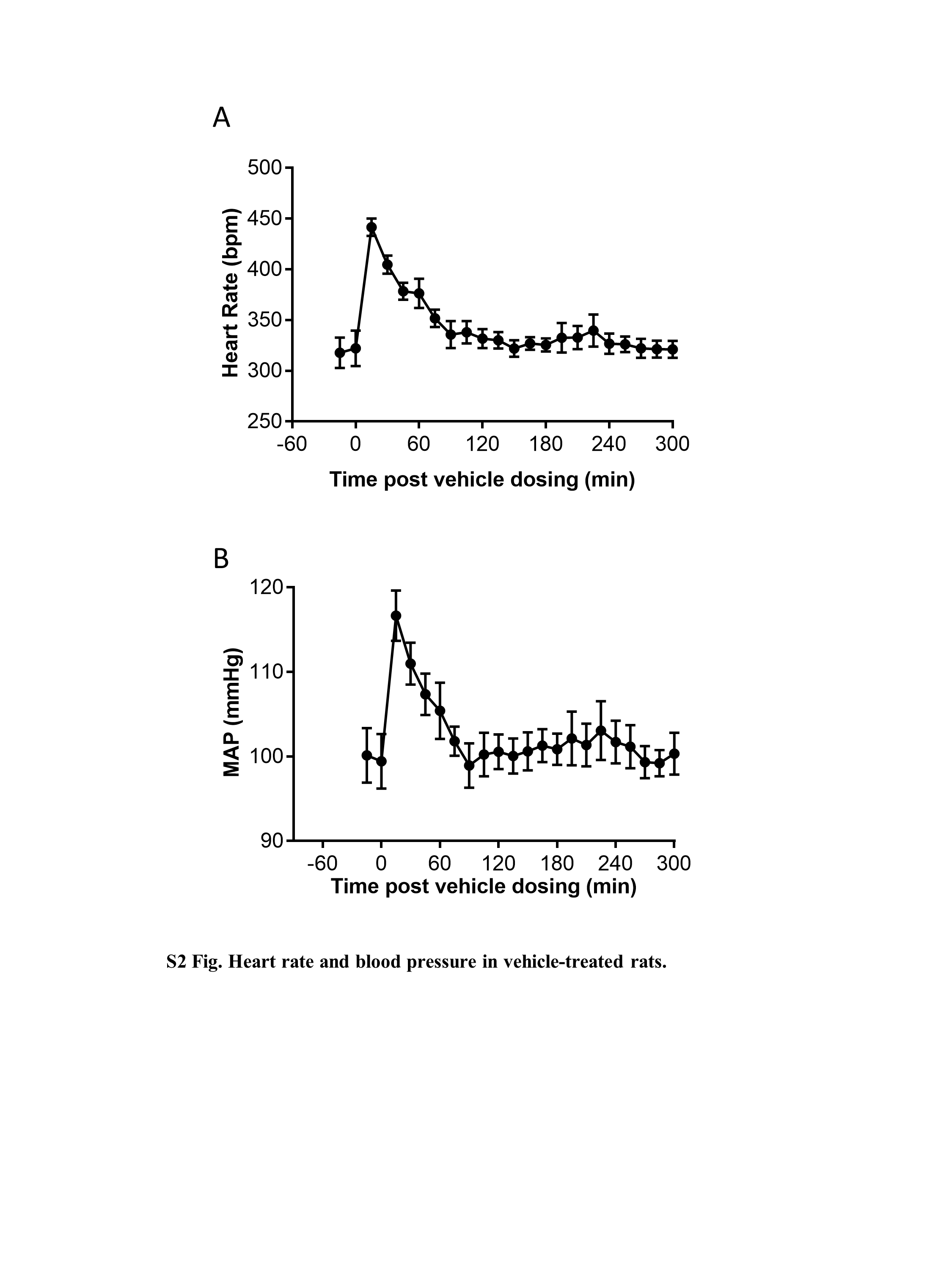

Supplement: S2 Fig — Heart rate (A) and mean arterial blood pressure (B) were measured immediately before and after intravenous administration of vehicle in telemeter-implanted rats that were housed in metabolic cages. Heart rate and blood pressure were analyzed separately using PROC GLM procedure for analysis of variance (ANOVA). The model included animal and post dosing time (min) as fixed categorical factors. Comparisons to 0 min (i.e. baseline) were performed to understand the vehicle effect. (TIF) [file pone.0202182.s002.tif]

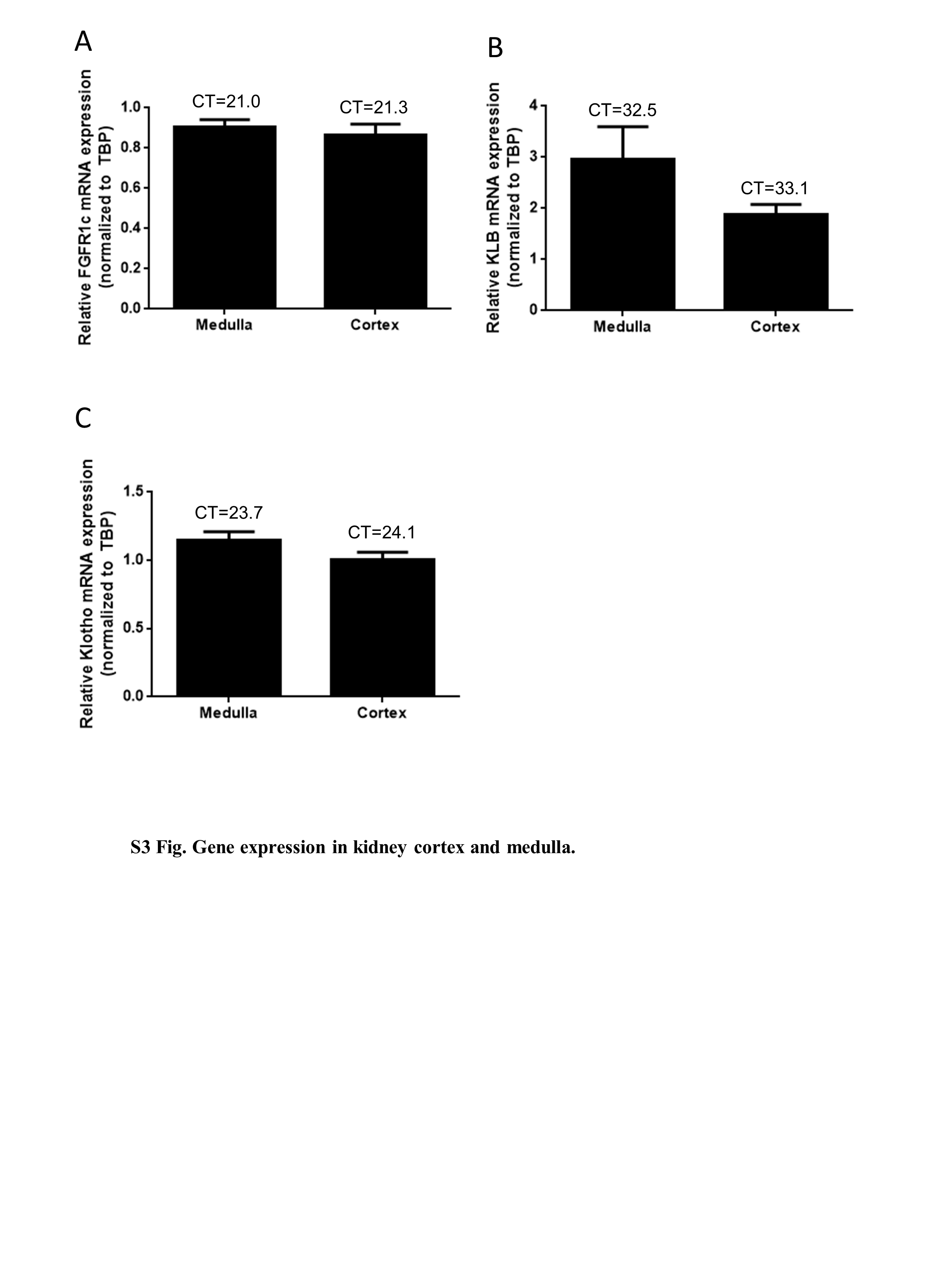

Supplement: S3 Fig — Gene expression of FGFR1c (A), KLB (B) and klotho (C) was assessed in the kidney cortex and medulla of vehicle-treated rats (n = 3). Data presented as mean ± SEM with Ct values shown for each dataset. Student t-test (unpaired, two tails) was utilized to determine significance. (TIF) [file pone.0202182.s003.tif]
