# Supplementary material for: FGF21 increases water intake, urine output and blood pressure in rats
Source: PLoS One. 2018 Aug 14;13(8):e0202182. doi: 10.1371/journal.pone.0202182 (PMC6091943; doi:10.1371/journal.pone.0202182)
Supplement: S1 Table — (DOCX) [file pone.0202182.s004.docx]

**S1 Table.** **Statistical information for results presented.**

| Figure | Test used | ANOVA F(DFn, DFd) |
| --- | --- | --- |
| 1A | 2-way repeated measure ANOVA | Interaction F(2, 54)=2.171  Time F(2, 54)=154.7  Group F(1, 54)=16.62 |
| 1B | 2-way repeated measure ANOVA | Interaction F(2, 34)=0.0102  Time F(2, 34)=1745  Group F(1, 17)=9.171 |
| 1C | 2-way repeated measure ANOVA | Interaction F(3, 72)=1.587  Time F(3, 72)=1132.7  Group F(1, 72)=0.002674 |
| 1D | 2-way repeated measure ANOVA | Interaction F(3, 72)=3.946  Time F(3, 72)=27.3  Group F(1, 72)=11.68 |
| 1E | 2-way repeated measure ANOVA | Interaction F(3, 72)=0.752  Time F(3, 72)=13.83  Group F(1, 72)=20.43 |
| 1F | 2-way repeated measure ANOVA | Interaction F(2, 51)=0.03504  Time F(2, 51)=0.06401  Group F(1, 51)=16.18 |
|  |  |  |
| 2A | 2-way repeated measure ANOVA | Interaction F(2, 51)=0.2813  Time F(2, 51)=0.03252  Group F(1, 51)=27.5 |
| 2B | 2-way repeated measure ANOVA | Interaction F(2, 34)=4.236  Time F(2, 34)=22.86  Group F(1, 17)=7.846 |
| 2C | 2-way repeated measure ANOVA | Interaction F(2, 34)=0.8752  Time F(2, 34)=1.054  Group F(1, 17)=3.811 |
| 2D | 2-way repeated measure ANOVA | Interaction F(2, 34)=0.2373  Time F(2, 34)=6.875  Group F(1, 17)=1.666 |
| 2E | 2-way repeated measure ANOVA | Interaction F(2, 18)=3385  Time F(1, 9)=1,453  Group F(2, 18)=5.915 |
|  |  |  |
| 3A | One-way ANOVA | F(3, 36)=86.79 |
| 3B | One-way ANOVA | F(3, 36)=8.279 |
| 3C | One-way ANOVA | Na F(3, 36)=32.17  K F(3, 36)=19.93  Cl F(3, 36)=23.25 |
| 3D | One-way ANOVA | Na F(3, 36)=4.24  K F(3, 36)=22.56  Cl F(3, 36)=15.86 |
|  |  |  |
| 4A | General Linear Model | Model DF=18, F=24.92  Animal ID DF=8, F=46.87  Time DF=10, F=7.36 |
| 4B (MAP) | General Linear Model | Model DF=18, F=23.75  Animal ID DF=8, F=38.85  Time DF=10, F=11.67 |
| 4B (DAP) | General Linear Model | Model DF=18, F=25.09  Animal ID DF=8, F=47.09  Time DF=10, F=7.5 |
| 4B (SAP) | General Linear Model | Model DF=18, F=19.68  Animal ID DF=8, F=28.17  Time DF=10, F=12.88 |
| 4C (Urine) | General Linear Model | Model DF=18, F=13.1  Animal ID DF=8, F=24.47  Time DF=10, F=4.01 |
| 4C (Water) | General Linear Model | Model DF=18, F=21.44  Animal ID DF=8, F=42.07  Time DF=10, F=4.93 |
| 4D | Unpaired Two-tailed T-test | T=0.2318 df=16 |
|  |  |  |
| 5A | Paired Two-tailed T-test | T=3.76, df=8 |
| 5B | Paired Two-tailed T-test | T=1.268, df=8 |
| 5C | Paired Two-tailed T-test | T=2.558, df=8 |
|  |  |  |
| 6B | Unpaired Two-tailed T-test | T=6.412 df=19 |
| 6C | Unpaired Two-tailed T-test | T=3.429 df=19 |
| 6D | Two-way ANOVA | Interaction F(1, 38)=0.08627  Treatment F(1, 38)=20.48  Group F(1, 38)=0.126 |
| 6E | Two-way ANOVA | Interaction F(1, 38)=0.0002485  Treatment F(1, 38)=6.034  Group F(1, 38)=1.579 |
| 6F | Two-way ANOVA | Interaction F(1, 41)=1.342  Treatment F(1, 41)=1.342  Group F(1, 41)=100.9 |
